# Integrated monitoring and evaluation and environmental risk factors for urogenital schistosomiasis and active trachoma in Burkina Faso before preventative chemotherapy using sentinel sites

**DOI:** 10.1186/1471-2334-11-191

**Published:** 2011-07-12

**Authors:** Artemis Koukounari, Seydou Touré, Christl A Donnelly, Amadou Ouedraogo, Bernadette Yoda, Cesaire Ky, Martin Kaboré, Elisa Bosqué-Oliva, María-Gloria Basáñez, Alan Fenwick, Joanne P Webster

**Affiliations:** 1 Schistosomiasis Control Initiative, Department of Infectious Diseases Epidemiology, St Mary's campus, Faculty of Medicine, Imperial College London, UK; 2 MRC Centre for Outbreak Analysis and Modelling, Department of Infectious Disease Epidemiology, Imperial College London, London, UK; 3 Programme National de Lutte contre la Schistosomiase et les Vers Intestinaux, Ministère de la Santé, Burkina Faso; 4 Programme National de Prevention de la Cecité, Burkina Faso; 5 Department of Infectious Disease Epidemiology, Imperial College London, London, UK

## Abstract

**Background:**

Over 1 billion of the world's poorest inhabitants are afflicted by neglected tropical diseases (NTDs). Integrated control programmes aimed at tackling these debilitating NTDs have been recently initiated, mainly using preventative chemotherapy. Monitoring and evaluation (M&E) of these integrated programs presents particular challenges over and above those required for single disease vertical programmes. We used baseline data from the National NTD Control Programme in Burkina Faso in order to assess the feasibility of an integrated survey design, as well as to elucidate the contribution of environmental variables to the risk of either *Schistosoma haematobium*, trachoma, or both among school-aged children.

**Methods:**

*S. haematobium *infection was diagnosed by detecting eggs in urine. A trachoma case was defined by the presence of Trachomatous inflammation-Follicular (TF) and/or Trachomatous inflammation-Intense (TI) in either eye. Baseline data collected from 3,324 children aged 7-11 years in 21 sentinel sites across 11 regions of Burkina Faso were analyzed using simple and multivariable hierarchical binomial logistic regression models fitted by Markov Chain Monte Carlo estimation methods. Probabilities of the risk of belonging to each infection/disease category were estimated as a function of age, gender (individual level), and environmental variables (at sentinel site level, interpolated from national meteorological stations).

**Results:**

Overall prevalence at the sentinel sites was 11.79% (95% CI: 10.70-12.89) for *S. haematobium*; 13.30% (12.14-14.45) for trachoma and 0.84% (0.53-1.15) for co-infections. The only significant predictor of *S. haematobium *infection was altitude. There were significant negative associations between the prevalence of active trachoma signs and minimum temperature, and air pressure. Conditional upon these predictors, these data are consistent with the two pathogens being independent.

**Conclusions:**

Urogenital schistosomiasis and trachoma constitute public health problems in Burkina Faso. Sentinel site (at school level) surveys for these two NTDs can be implemented simultaneously. However, to support MDA treatment decisions in Burkina Faso, the protocol used in this study would only be applicable to hypoendemic trachoma areas. More research is needed to confirm if these findings can be generalized to West Africa and beyond.

## Background

Schistosomiasis and trachoma are among the most prevalent of the so-called "Neglected Tropical Diseases (NTDs)", an umbrella term that encompasses a group of parasitic, bacterial, and viral infections collectively imposing a similar disease burden to that of malaria and HIV [1,2]. NTDs are widespread in sub-Saharan Africa [3], where they affect more than 500 million people [1]. These NTDs are both the result of, and a major contributor to the poverty of many rural and some disadvantaged urban populations in tropical regions of the world [4]. However, the fact that some NTDs are treatable with affordable (or donated), safe and effective drugs has encouraged the implementation of 'preventative' chemotherapy control programs. Although these drugs do not prevent such infections, they help prevent or minimize the burden of disease that would ensue if left untreated.

According to the World Health Organization (WHO), more than 206 million people are infected with parasites of the genus *Schistosoma *(of whom 120 million have symptoms and around 20 million show severe disease sequelae) [5]. Schistosomiasis is an indirectly (snail)-transmitted disease whose distribution is particularly sensitive to environmental changes, including changes of human origin. Transmission of the parasite is highly focal, with heterogeneity reflecting numerous human and snail host as well as environmental factors [6]. Identifying the broad scale patterns of schistosomiasis is crucial because ecological and climatic changes plus human migration may have led to changes in the prevalence and distribution of the disease in different parts of endemic countries [7]. The current mainstay for schistosomiasis control is to mitigate against the burden of the disease by controlling morbidity through chemotherapy with praziquantel (PZQ). It is recommended that mass drug administration (MDA) with PZQ be delivered to total at-risk populations defined where a survey shows prevalence of over 50% in school-aged children and to children aged 6-16 years where survey prevalence is between 10% and 50% in this group [8]. (Although the indicator group for schistosomiasis comprises school-aged children, pre-school-aged children can also be infected, the implications of which are considered below). Because schistosomiasis exhibits strong spatial heterogeneity at local levels, there is a need to identify and locate high-risk communities or schools that require MDA [7] so that cost-effective management of limited resources can be achieved.

Trachoma is the leading infectious cause of blindness worldwide [9]. Around 80 million people have active trachoma, an inflammatory early stage of the disease caused by recent infection with the ocular bacteria *Chlamydia trachomatis*, with the young children bearing the heaviest burden [10]. Repeat infection leads to scarring of the eyelid, which causes the eyelids to turn inwards and subsequently cause the eyelashes to scratch the cornea, ultimately leading to blindness (WHO estimates that 6 million people globally are blind from trachoma). The WHO endorses a four-pronged approach to eliminate blinding trachoma by the year 2020 known as SAFE: Surgery for trichiasis, Antibiotic against infection, Facial cleanliness, and Environmental sanitation [11]. Currently, Pfizer Inc. donates millions of doses of the antibiotic azithromycin (Zithromax, ZTX) for trachoma control. MDA with ZTX or tetracycline eye ointment is at present recommended for entire endemic communities to be conducted annually for three years wherever the prevalence of active trachoma in children of ages 1-9 years old at the district level is above 10% [11]. Trachoma is associated with individual and environmental risk factors as well as climatic conditions which may be important determinants of trachoma transmission (which in some cases can occur mechanically through flies) [12-15]. These may vary between settings and hence the need for studies aimed to identify risk factors relevant to specific environments. Indeed, understanding risk factors is essential in designing appropriate interventions for the 'F' and 'E' components of the SAFE strategy (e.g. water availability for Facial cleanliness and Environmental improvements) [16].

Based on the argument of co-endemicity of NTDs within countries [17], global advocacy for the amelioration or elimination of the disease burden imposed by these conditions has recently increased, with a renewed interest in the scaling-up of integrated NTD interventions in sub-Saharan Africa, mainly using preventative chemotherapy distributed through MDA [18]. More particularly, MDA is a relatively efficacious tool to control the infection and morbidity caused by the helminthiases and trachoma and such an approach offers opportunities for integration. There is also an interest in trying to deliver some of treatments through school-based programmes, and again schistosomiasis and trachoma lend themselves to this approach as the burden of infection is mostly concentrated among the young.

An important question in integrated NTD control relates to the spatial scale at which the programs should be integrated. This will depend on the geographical distribution and prevalence of both single and co-infections. Therefore, the principal aim of the current study was to identify whether combined survey designs based on sentinel sites are useful monitoring and evaluation (M&E) tools for the assessment of integrated schistosomiasis and active trachoma control programmes. A secondary aim was to establish the distribution of these two NTDs in these selected sentinel sites within Burkina Faso pre- and post-treatment (post-treatment data are not presented here). Finally, a post hoc analysis studied associations of urogenital schistosomiasis infection and active trachoma disease with demographic and environmental variables; such results should inform the construction of reliable disease maps [19] and mathematical modeling efforts of these two NTDs. In addition, these results should help highlight potential factors related to the physiology and behavior of mechanical vectors of these two NTDs which ultimately could inform policies aimed at reducing transmission of these diseases.

## Methods

### Environmental conditions, control programme, sentinel sites and sampling

In September 2006, the United States Agency for International Development (USAID) launched the NTD Control Program, the first global effort to support country programs to integrate and scale-up delivery of preventative chemotherapy for five targeted NTDs: schistosomiasis, trachoma, lymphatic filariasis (LF), onchocerciasis and soil-transmitted helminthiasis (STHs). Burkina Faso, a landlocked country in West Africa, is endemic for all these five NTDs [20] and thus it was selected as one of the first five country programs for implementation of integrated NTD control. The, environmental factors that influence disease transmission are unlikely to be uniform over large geographical areas [21]. Consequently, when designing integrated control programs, it is essential to know the distribution and abundance of the target diseases, to enable optimizing both the targeting of suitable interventions and the use of available resources [22]. Currently, however, there is little evidence on the feasibility of integrated survey designs [23].

Burkina Faso has a tropical climate with two very distinct seasons: the rainy season with between 600 and 900 mm of rainfall, and the dry season during which the Harmattan, a hot dry wind from the Sahara, blows. The rainy season lasts approximately four months, May/June to September, and is shorter in the northern part of the country. Four large agro-climatic regions can be defined, characterized by a strong south-north decreasing gradient of average annual rainfall. The Sahel in the north receives less than 500 mm rainfall a year and exhibits high temperatures [24].

Although initially control programmes for schistosomiasis, trachoma, LF, onchocerciasis and the STHs operated autonomously, the Burkina Faso NTD Control Program has recently taken a phased approach to integrated MDA. For instance, Burkina Faso was under the umbrella of the Onchocerciasis Control Programme in West Africa until 2002 and community-directed treatment with ivermectin (CDTI) is the mainstay of control in areas where the infection was not eliminated. For the case of schistosomiasis and trachoma, in 2007 drugs were distributed for these two infections and STHs as an integrated package in three target districts (Tenkodogo, Koupela and Bogandé) and in 2008 these efforts were scaled up to include the LF and onchocerciasis programmes as a platform for becoming a fully integrated MDA campaign on a national level. At the time these surveys were planned, integrated assessments of schistosomiasis and trachoma as well as their co-infections using sentinel sites were made the main focus of these efforts.

In the current study we used baseline data (collected during November 2007 and February 2008 from 21 sentinel sites across 11 regions of the country) from longitudinal surveys which aimed also to contribute to the assessment of the impact on schistosomiasis and trachoma disease burden of the integrated NTD control programme in Burkina after MDA (i.e. once the follow-up data would be collected a year later). We will present the analyses of the follow-up studies elsewhere.

Sentinel sites were schools which were chosen based on a list which encompassed villages to be endemic for onchocerciasis under the surveillance of the African Programme for Onchocerciasis Control (APOC). Among these villages, those which were also endemic for LF and schistosomiasis (based on national historical data at the time) were selected randomly. Then, once at the sentinel site, the method for selecting school children in each of the ages of the 7-11 years olds, was random sampling. An M&E meeting between the National Coordinators and Schistosomiasis Control Initiative (SCI) staff preceded these surveys in February 2007 in Ouagadougou in order to decide on the cohort design and sampling.

A fixed cohort was recruited at each sentinel site to allow comparisons to be made across sentinel sites and regions. Required sample sizes were calculated using the EpiSchisto software tool http://www.schoolsandhealth.org/Pages/EpiDynamics.aspx based on an expected reduction in mean infection intensity of 40% (for *S. haematobium*) following chemotherapy to achieve 80% statistical power and a significance level of 5%. The value of 40% was chosen as a conservative estimate of the expected reduction over a two-year period (two annual PZQ treatments-see Additional file 1, section S1, Figures S1A and S1B, for further details concerning sample size calculations). A cluster random sampling design was not considered appropriate here (despite the fact that this is the most reliable survey method for trachoma prevalence estimation) as one of the purposes of this study was to utilize an integrated method [25]. Detailed statistical information has been provided in previous studies [20,26]. All children enrolled in the study were interviewed by appropriately trained personnel at the Ministry of Health, Burkina Faso, after parental consent was acquired. Administrative and religious authorities, among others, were all involved in these surveys in order to facilitate the implementation in the field. Ethical clearance was obtained from the Burkina Faso Ministry of Health and Imperial College London.

### Indicators for urogenital schistosomiasis

Experienced technicians from the National Vertical Schistosomiasis Control Programme (NVSCP) collected from the children one urine specimen to determine the prevalence and intensity of *S. haematobium *infection by the filtration method. The intensity of *S. haematobium *infection was expressed as the number of eggs per 10 mL of urine.

### Indicators for active trachoma

Clinical signs of active trachoma, namely, trachomatous inflammation-follicular (TF) and trachomatous inflammation-intense (TI), were graded for each eye separately by two ophthalmologists and an ophthalmic nurse according to the WHO simplified grading system [27]. Standardization of the grading by the examiners and thus reliability tests were not performed during the baseline surveys but they did happen before the follow-up studies. In this study, active trachoma was taken to indicate trachoma signs, and was defined as the presence of either of these signs (TF, TI) in either eye. All children with evidence of active trachoma were treated according to national guidelines and advice was then given on face washing. The nurse cleaned his/her hands with alcohol and the examiner changed gloves after examining the eyes of each child.

### Interpolation of environmental data, exploratory and statistical analysis

Environmental information from all nine meteorological stations in Burkina Faso were obtained on a daily basis for 2000 to 2008 for the following variables: altitude (in meters above sea level - MSL), precipitation (in millimeters - mm), minimum, maximum, and average temperature (in degrees Celsius-°C) as well as air pressure (in millibars- mbars) from the National Climatic Data Center (NCDC) website http://www7.ncdc.noaa.gov/CDO/cdoselect.cmd?datasetabbv=GSOD&countryabbv=&georegionabbv=. Such variables were selected based on their potential biological relevance to the intermediate hosts of schistosomiasis and the mechanical (fly) vectors of trachoma. The average values over these years for each of these environmental variables at each meteorological station were then calculated. Similar data were not available for the twenty one sentinel sites of this study individually but their geographical positions were recorded using Garmin^® ^GPS devices.

Inverse distance weighted (IDW) interpolation was employed to estimate such variables for the sentinel sites, by using a weighted sum of the values of the nine known points for each environmental variable. More precisely, IDW assigned weights to sample points (i.e. sentinel sites), which are proportional to the inverse distance squared that the particular school sentinel site is separated from the point of estimation (i.e. the meteorological station). The equation for the inverse squared distance weighted interpolation used, is as follows:

where *d_i _*is the distance between the sentinel school site and weather station *i*. The *u_i _*term is the average values of environmental variables as calculated for meteorological station *i*. This approach makes use of the spatial autocorrelation in the environmental data because only points relatively close to the point of estimation will affect the prediction.

Exploratory analyses were undertaken to explore the structure of the datasets: Firstly, Pearson correlations were calculated for the average values of the environmental variables at each meteorological station (Additional file 1 section S2, Table S1). Secondly, Pearson correlations between the school-level prevalences of single and dual infections with the interpolated environmental variables were calculated (Additional file 1, section S2, Table S2). Thirdly, scatter plots were produced to examine relationships between school-level prevalences of single and dual infections with the interpolated environmental variables (Additional file 1, section S3). Finally, summary statistics were generated for the environmental variables (Table 1).

**Table 1:** Summary statistics of interpolated and meteorological station-based variables

Environmental & weather variables	Interpolated (i.e. for sentinel sites; n = 21)					At weather stations (n = 9)			
	**Mean**	**Median**	**Min**	**Max**	**S.D**.	**Mean**	**Median**	**Min**	**Max**
Altitude*	321.1	304.0	259.0	440.8	52.0	319.7	297.0	259.0	456.0
**Precipitation**^◊^	2.37	2.39	1.53	2.98	0.40	2.43	2.52	1.53	2.98
**Min temperature**^‡^	22.8	22.9	21.8	23.3	0.3	22.8	22.8	21.8	23.4
**Max temperature**^♦^	35.5	35.6	34.0	37.3	0.9	35.4	35.4	33.8	37.3
**Av temperature**^‡^	28.7	28.7	27.5	29.5	0.4	28.6	28.6	27.5	29.5
**Air pressure**^▲^	973.5	975.1	961.0	978.5	4.8	973.9	975.8	959.0	979.2

*MSL: Meters above sea level; ^◊ ^mm: millimeters; ^♦^°C: Celsius; ^‡°^C: Celsius; ^▲ ^mbars: millibars

For the modeling of the relationship between the *S. haematobium *prevalence and the interpolated environmental variables, binomial logistic regression models were used. Similar models were applied in order to examine the relationship between active trachoma prevalence and the interpolated environmental variables. Potential confounding effects (age and gender) were adjusted for in all logistic regression models. A problem in developing models using environmental variables is that many such variables are highly inter-correlated making it difficult to separate the effects of the independent variables statistically [28]. To reduce the dimensionality of these collinear variables, firstly we selected those variables likely to have greater biological significance on infection transmission [29]. To take into account the nested structure of our data we fitted hierarchical models with Markov Chain Monte Carlo (MCMC) estimation methods, including schools as random effects. Children within each school as well as the results from different schools were treated as 'exchangeable' in the sense that each pair of children within a school were equally correlated [30]. We used the MLwiN v 2.21 default setting which specify diffuse prior distributions (fixed parameters *p*(*β*) ∝ 1 and scalar variances  and which approximate maximum likelihood estimates. The additional variables were included in the models following a backward stepwise elimination procedure, comparing the deviance information criterion (DIC) at each stage [31]. We considered as potential explanatory variables the interpolated values of altitude, precipitation, minimum temperature, maximum temperature, average temperature, and air pressure at all sentinel sites and we calculated 95% credible intervals (i.e. intervals derived from 2.5% and 97.5% quantiles of the chains of parameter estimates). Odds ratios from all these models were reported relating to differences comparable to rounded standard deviations of the interpolated environmental variables (see Table 2) to make the results more readily interpretable.

**Table 2:** Observed prevalence by type of infection (urogenital schistosomiasis and trachoma) and sentinel site in 21 schools of Burkina Faso)

Prevalence of type of infection by sentinel site with Exact Binomial 95% Confidence Intervals (n = 3,324 children aged 7-11 years)			
	Only *S. haematobium *infection	Only trachoma signs	Co-infection with trachoma signs and *S. haematobium*
**Sentinel site** **(size)**			
**BADONGO (n = 148)**	20.95 (14.70 - 28.39)	12.84 (7.91 - 19.32)	0 (0.00 -2.46)
**BAWAN** **(n = 203)**	2.96 (1.09 - 6.32)	12.81 (8.54 - 18.20)	0.49 (0.00 - 2.71)
**BAYANDI PALOGO** **(n = 141)**	3.55 (1.16 - 8.08)	7.09 (3.45 - 12.66)	0 (0.00 - 2.58)
**DOUNA** **(n = 219)**	17.35 (12.58 -23.03)	4.57 (2.21 - 8.24)	0 (0.00 -1.67)
**GORA** **(n = 211)**	0 (0.00 -0.02)	13.27 (9.00 - 18.61)	0 (0.00 -0.02)
**KARI** **(n = 224)**	0.45 (0.00 - 2.46)	20.54 (15.44 - 26.42)	0.45 (0.00 - 2.46))
**KOUMBRI** **(n = 156)**	19.23 (13.37 - 26.30)	5.77 (2.67 - 10.67)	0.64 (0.00 -3.52)
**LERBOU** **(n = 91)**	45.05 (34.60 - 55.84)	0 (0.00 - 3.97)	3.30 (0.00 -9.33)
**LIOULGOU** **(n = 147)**	9.52 (5.31 - 15.46)	10.88 (6.35 - 17.07)	0.68 (0.00 - 3.73)
**MEDIGA** **(n = 150)**	27.33 (20.38 - 35.20)	9.33 (5.20 - 15.16)	3.33 (1.09 -7.61)
**MINIMA DOURE** **(n = 99)**	14.14 (7.95 - 21.59)	2.02 (0.25 - 7.11)	2.02 (0.25 - 7.11)
**NAGBINGOU** **(n = 146)**	20.55 (14.31 - 28.02)	8.22 (4.32 - 13.92)	1.37 (0.17 - 4.86)
**NOUMOUSSO** **(n = 218)**	0 (0.00-1.68)	32.57 (26.39 -39.23)	0.46 (0.00 - 2.53)
**PANAMASSO** **(n = 216)**	0 (0.00 - 1.69)	35.65 (29.27 - 42.43)	0 (0.00 - 1.69)
**SAMPIERI** **(n = 121)**	5.79 (2.36 - 11.56)	10.74 (5.85 - 17.67)	0 (0.00 - 0.03)
**SIDOGO** **(n = 153)**	7.84 (4.12 -13.30)	9.80 (5.59 - 15.65)	0.65 (0.00 - 3.59)
**SOALA** **(n = 148)**	9.46 (5.27 - 15.36)	12.84 (7.91 - 19.32)	4.05 (1.50 - 8.61)
**TIAO** **(n = 131)**	0 (0.00 - 2.78)	6.87 (3.19 - 12.64)	0 (0.00 - 2.78)
**TIKAN** **(n = 147)**	10.20 (5.82 - 16.27)	2.72 (0.75 - 6.82)	0.68 (0.00 -3.73)
**TOUGOURI** **(n = 136)**	14.71 (9.22 - 21.79)	9.56 (5.19 - 15.72)	0.74 (0.00 - 4.03)
**WINDOU** **(n = 119)**	37.82 (29.09 - 47.16)	0.84 (0.00 - 4.59)	1.68 (0.00 - 5.94)

Finally, we used the map-making facility available at http://www.spatialepidemiology.net, which utilizes Google Maps [32], in order to display the NCDC weather stations and sentinel sites visited during the M&E surveys in Burkina Faso. We also used this tool to display the observed *S. haematobium *and active trachoma prevalence and treatment strategy, as well as the need for intervention with the SAFE strategy, based on WHO-recommended implementation thresholds [33]. It should be noted, however, that although for trachoma these thresholds were established based on prevalence of clinical signs of disease in children 1-9 years of age, the observation that the prevalence in school-aged children is greater than 10% probably means that this prevalence is even greater in the younger age groups, warranting treatment.

## Results

**Figure 1:**
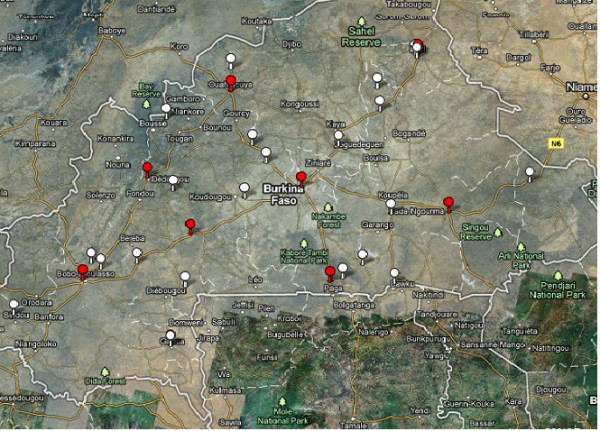
**Location of sentinel sites (white markers) and meteorological stations (red markers) in Burkina Faso for the assessment of urogenital schistosomiasis and active trachoma in school-based surveys**.

A total of 3,324 children aged 7 to 11 years were surveyed from the 21 sentinel sites (Figure 1). Observed prevalence values by sentinel site of single and dual *S. haematobium *and trachoma signs are illustrated in Table 2.

**Figure 2:**
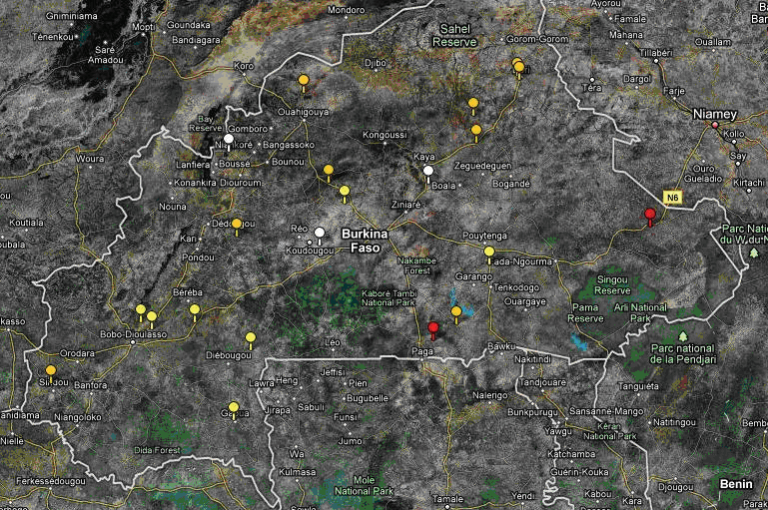
**MDA recommendations for schistosomiasis and trachoma for the 21 sentinel sites from observed data on prevalence of the two diseases; low *S. haematobium *prevalence (< 10%) and low trachoma prevalence (< 10%) (white markers; no MDA recommended); low *S. haematobium *prevalence (< 10%) and high trachoma prevalence (> 10%) (yellow markers; MDA for trachoma is recommended); medium *S. haematobium *prevalence (10-49%) and high trachoma prevalence (> 10%) (red markers; MDA in school-aged children; community MDA for trachoma)**.

Figure 2 illustrates MDA recommendations for *S. haematobium *and trachoma signs for the 21 sentinel sites from observed data on the prevalence of the two diseases. Our data suggest that in only two sentinel sites would both PZQ and ZTX be required. In addition, Figure 2 shows that to the north (i.e. in the driest areas) of Burkina Faso the prevalence of *S. haematobium *infection was between 10% and 49% (i.e. medium) but that of trachoma was less than 10% (i.e. low).

Table 2 contains summary statistics for the interpolated and meteorological station-based values of the environmental variables. For instance, for altitude, minimum (min) and maximum (max) values were 259.0 and 440.8 MSL respectively; for precipitation, min and max values were 1.53 and 2.98 mm; for max temperature, min and max values were 34.0 and 37.3°C: for min temperature, min and max values were 21.8 and 23.3°C; for average temperature, min and max values were 27.5 and 29.5°C and finally for air pressure, min and max values were 961.0 and 978.5 mbars. The distributions of the interpolated values appear to be similar to the distributions of the original station-based data.

Table 3 contains the odds ratios (ORs) and 95% credible intervals (95% CrI) of the significant environmental variables for the risk of *S. haematobium *infection as being estimated from the simple and multivariable hierarchical logistic regression models. According to simple hierarchical logistic regression modelling, the risk of *S. haematobium *infection was significantly increased for higher temperatures (minimum, maximum and average); higher air pressure; lower altitude; and lower precipitation. Such associations were also noticeable in the scatter plots (Additional file 1, section S3). A multivariable hierarchical logistic regression model was then fitted (without including maximum and average temperatures due to collinearity with minimum temperature). The risk of *S. haematobium *infection was indicated to be only negatively and significantly associated with altitude; a 50 MSL increase in altitude would decrease the odds of *S. haematobium *infection by 99.8%.

**Table 3:** Odds Ratios and 95% credible intervals for the risk of *S. haematobium *infection (n = 3,324)-adjusted for host age and gender in 21 school sentinel sites in Burkina Faso

	Simple hierarchical logistic regression models	Final multivariable hierarchical logistic regression model
Environmental and weather variables	ORs (95% Credible Intervals)	ORs (95% Credible Intervals)
Altitude*	0.168 (0.118 - 0.242)	0.168 (0.118 - 0.242)
**Precipitation**^◊^	0.406 (0.322 - 0.510)	REM
**Min temperature**^‡^	2.856 (2.686 - 3.000)	REM
**Max temperature**^♦^	2.313 (2.198 - 2.385)	NI
**Av temperature**^‡^	1.742 (1.710 - 1.779)	NI
**Air pressure**^▲^	2.151 (2.140 - 2.174)	REM

NI: These variables were not included in the final multivariate binomial logistic regression model building, as DIC indicated poorer fit than when min temperature was included.

REM: The effects of these variables were removed as DIC indicated by backward elimination

*per 50 MSL: Meters above sea level; ^◊^per 0.5 mm: millimeters; ^♦^per 1°C: Celsius; ^‡ ^per 0.5°C: Celsius; ^▲^per 5 mbars: millibars

Table 4 contains the ORs and 95% CrI of the significant environmental variables for the risk of trachoma signs as being estimated from the simple and multivariable hierarchical logistic regression models. Simple hierarchical logistic regression models indicated (perhaps counter-intuitively) an increased risk of trachoma signs at higher altitudes and greater precipitation, and a decreased risk of trachoma signs at higher temperatures (minimum, maximum, and average), and air pressure. On inspection of the scatter plots of active trachoma prevalence by the environmental variables, such associations were noticeable (Additional file 1, section S3). Similarly as before, maximum and average temperatures were excluded from the multivariable hierarchical logistic regression model building due to collinearity with minimum temperature. On multivariable analysis, the risk of trachoma signs remained decreased at higher minimum temperature and higher air pressure. Altitude and precipitation were finally removed from the final multivariable hierarchical logistic regression on the inspection of credible intervals and the DIC, through the backward elimination method.

**Table 4:** Odds Ratios and 95% credible intervals for the risk of trachoma signs (n = 3,324)-adjusted for host age and gender in 21 school sentinel sites in Burkina Faso

	Simple hierarchical logistic regression models	Final multivariable hierarchical logistic regression model
Environmental and weather variables	ORs (95% Credible Intervals)	ORs (95% Credible Intervals)
Altitude*	1.730 (1.189 - 2.433)	REM
**Precipitation**^◊^	1.690 (1.336 - 2.016)	REM
**Min temperature**^‡^	0.570 (0.546 - 0.591)	0.746 (0.717 - 0.768)
**Max temperature**^♦^	0.465 (0.436 - 0.484)	NI
**Av temperature**^‡^	0.580 (0.564 - 0.591)	NI
**Air pressure**^▲^	0.606 (0.602 - 0.609)	0.616 (0.608 - 0.622)

NI: These variables were not included in the final multivariate binomial logistic regression model building as DIC indicated poorer fit than when min temperature was included.

REM: The effects of these variables were removed as DIC indicated by backward elimination

*per 50 MSL: Meters above sea level; ^◊ ^per 0.5 mm: millimeters; ^♦ ^per 1°C: Celsius; ^‡ ^per 0.5°C: Celsius; ^▲ ^per 5 mbars: millibars

## Discussion

Through sentinel site surveillance defined at the school level, this study showed that urogenital schistosomiasis and trachoma surveys can be successfully implemented simultaneously. In addition, the prevalence of schistosomiasis infection and trachoma signs in these sentinel sites before integrated treatment in Burkina Faso was estimated. Results indicated that both of these diseases are prevalent in the country and they likely do constitute public health problems according to WHO recommended thresholds [15,20,34-36]. Hierarchical binomial logistic regressions identified, as may be predicted, these diseases to be independent (conditional on environmental factors), which further highlight the different mechanisms of their transmission. Treatment with both PZQ (for urogenital schistosomiasis) and ZTX (for trachoma) was only required, however, for two of the 21 sentinel sites surveyed here.

We chose sentinel site surveillance conducted in schools because in the case of schistosomiasis the selected methodology is ideal for the following reasons [37]: (i) schools are accessible and receptive; (ii) the highest prevalence levels of *S. haematobium *infection are found among school-age children; (iii) data collected in this age range may be used to evaluate not only if schistosomiasis threatens the health of school-aged children, but also if there is need for intervention in the community as a whole; (iv) children in intermediate grades (generally between ages 9-12) allow for the accompaniment of treatment impact over one to two years, before they leave school. In addition, we chose to recruit a longitudinal cohort of 7-11 years old at baseline for both logistical reasons (through integrating with the ongoing surveys) and as this would enable us to follow-up these same children during three years. Furthermore, our past experience with SCI data has demonstrated that morbidity is also detectable in young school-aged children, which suggests that children may become infected early in life [20,38,39]. As we were interested ultimately to monitor changes in the infection and to quantify the rate at which infections change, we suspect that changes in morbidity in the younger age groups of school children would be more sensitive to changes in the force of infection (i.e. per capita rate at which a host acquires new infections) by MDA. However, we recognize that if we had also included data from the even younger age groups (i.e. pre- school children) who did not receive schistosomiasis treatment in the monitoring programme, our understanding of the secular changes in transmission caused by a morbidity control programme could be improved, helping also differentiate between the impact of PZQ on the individual and the community [40].

School-based surveys might underestimate the prevalence of trachoma in the community as a whole and they certainly do not determine the priority for surgical intervention [25]. In addition, a key feature of trachoma epidemiology is that of the age-profile of infection prevalence, which increases to a peak at very young ages and declines at older ages (particularly in heavily infected communities). This feature would not be captured in study designs such as that used in the present study [16,41-43]. Trachoma interventions using WHO thresholds were established for a different age group than the one analyzed here and thus we are aware that inference on this matter should be treated with some caution. Nevertheless, as the distribution of infection and disease will vary according to endemicity levels, the protocol applied in this study could be of use to hypoendemic trachoma areas of Burkina Faso if MDA treatment decisions have to be made based on results arising from similar study designs but not for planning where SAFE implementation is needed as this is based on district-level prevalence of TF among the preferred 1-9 year age group. We draw this conclusion because it has been observed in hypo- and meso-endemic trachoma areas the infection prevalence data come to a peak in ages slightly greater than 5 years [41].

In this school-based approach a significant proportion of the school-aged children may not attend school and so the practicality of this approach may be limited to areas where school attendance is not too low. In Burkina Faso the percentage of attendance for the primary schools (children aged 7-13 years old) is approximately 63% and thus we might have missed a significant proportion of *S. haematobium *infection and trachoma signs. Moreover, observer error might have compromised the sensitivity and specificity of clinical diagnosis, although the magnitude of this effect is likely to be smaller [44].

Environmental effects on the emergence and re-emergence of pathogens are well recognized and thus a secondary aim of this study was to characterize quantitatively the effect of specific environmental factors in addition to demographic factors on the two NTDs of interest here: urogenital schistosomiasis and trachoma.

Altitude, precipitation, temperature and air pressure were all important predictors of *S. haematobium *infection (Table 3) by simple hierarchical logistic regression modelling. Temperature and rainfall were important predictors of prevalence of *S. haematobium *infection also in Tanzania [45] and such statistical relationships are consistent with the known biology of freshwater snails, the intermediate hosts for schistosomes. *S. haematobium *is transmitted by snails from the genus *Bulinus*. In Burkina Faso *B. truncatus *and *B. senegalensis *occur in all endemic schistosomiasis areas of the country [35,46] and their dynamics are known to be sensitive to temperature and rainfall [29]. However, multivariable analysis indicated only altitude to be negatively significantly associated with prevalence of *S. haematobium *infection.

There were significant negative associations of prevalence of trachoma signs with minimum temperature and air pressure (Table 4). The significant negative association between minimum temperature as well as air pressure and trachoma signs in our study, strengthens the argument that environmental factors, and particularly climatic conditions, may be important determinants of the physiology and behavior of *Musca sorbens*, a mechanical vector of trachoma in some settings [10,13,15]. Even if these are speculative, the following mechanisms can also be envisaged [14]: Preferred breeding sites of *M. sorbens *are fresh human faecal materials lying on the ground. High temperature and sunshine may induce rapid dryness of faecal materials, causing them to become inadequate breeding sites. Moreover, the lifespan of *M. sorbens *strongly depends on temperature, decreasing from 35 days at 24°C to less than 12 days at 32°C, yielding less frequent contacts of *M. sorbens *with children's faces, and in turn, lower contamination of *M. sorbens *with *C. trachomatis *[47,48]. We did not find any significant association between age and trachoma prevalence and we suspect that this is due to the fact that pre-school aged children are not included in the current surveys. Conditional upon these predictors, these data are consistent with the two pathogens being independent.

As mentioned previously, the present study was designed to be as simple as possible in order to increase participation and to make it logistically manageable in settings with few resources. We therefore did not collect observational data, such as water-use behavior, evidence of latrine use and hygienic habits/states. Perhaps an investigation of the relationship between fly density, markers of socio-economic status, hygienic habits/states and altitude in these sentinel sites could be informative. Such facts may have led us to missing more subtle or complex relationships between risk factors for urogenital schistosomiasis and active trachoma [14] in the current study.

We believe that despite the potential limitations contained in terms of age groups selected for the assessment of *S. haematobium *infection and active trachoma, the fact that there was found very low prevalence of co-infections in the studied sentinel sites, carries important implications for integrated NTDs surveillance and control strategies. The presence of relatively few co-infections has clear consequences for the concept of integration of mapping, planning of drug delivery, and M&E of integrated control programs for these two NTDs. Results of the current study are in accordance with observations reported in the Plateau and Nasarawa States in Nigeria [17]. We agree with these authors' recommendation that current population-based district level surveys for the mapping of trachoma should not be replaced by school-based surveys. Finally, given that NTD interventions require baseline data for targeting treatment and evaluation, the benefits of including more than one disease indicator in overlapping age groups when using an integrated M&E protocol, warrants further study. In particular, different study designs would require further investigation. More research would also be needed to ascertain whether the findings presented here can be generalized to West Africa and beyond.

## Conclusions

To our knowledge, very few previously published studies-three in Nigeria, Southern Sudan and Togo [17,23,49] and another more recent one offering a conceptual framework around these issues [50]-have discussed and tested different survey methodologies for M&E of different NTDs in integrated control programmes. The current study aims to bridge some of these gaps and stimulate further research around the methodologies for M&E for different NTDs by reporting on baseline findings from sentinel site surveillance of *S. haematobium *and trachoma signs with an integrated M&E protocol in Burkina Faso. We conclude that the current approach may be useful to monitor and evaluate integrated programs including trachoma, but not for planning where SAFE implementation should occur. In addition, we would recommend that current methodology to be modified to assess the younger age groups both for schistosomiasis and trachoma so that literature gets enriched from such data in the case of schistosomiasis, and at the same time key features of trachoma epidemiology can be captured.

## Competing interests

The authors declare that they have no competing interests.

## Authors' contributions

AF obtained funding and AF & JPW were the principal investigators. AK, ST, CAD, BY, EBO and JPW, participated in the design of data collection. ST, AO, BY, CK and MK participated in data collection. AK drafted the manuscript. AK carried out statistical analysis. All authors contributed to the critical revision of the manuscript for important intellectual content and agreed on submission.

## Pre-publication history

The pre-publication history for this paper can be accessed here:

http://www.biomedcentral.com/1471-2334/11/191/prepub

## Supplementary Material

Additional file 1 **Supplement for the manuscript 'Integrated monitoring and evaluation and environmental risk factors for urogenital schistosomiasis and active trachoma in Burkina Faso before preventative chemotherapy using sentinel sites'**. This file contains some further statistical analysis that supports the methodology finally used. Click here for file
