# Supplementary material for: Integrated monitoring and evaluation and environmental risk factors for urogenital schistosomiasis and active trachoma in Burkina Faso before preventative chemotherapy using sentinel sites
Source: BMC Infect Dis. 2011 Jul 12;11:191. doi: 10.1186/1471-2334-11-191 (PMC3161883; doi:10.1186/1471-2334-11-191)
Supplement: Additional file 1 — Supplement for the manuscript 'Integrated monitoring and evaluation and environmental risk factors for urogenital schistosomiasis and active trachoma in Burkina Faso before preventative chemotherapy using sentinel sites'. This file contains some further statistical analysis that supports the methodology finally used. [file 1471-2334-11-191-S1.DOCX]

**Supplement for the manuscript ‘Integrated monitoring and evaluation and enviromental risk factors for urinary schistosomiasis and active trachoma in Burkina Faso before preventative chemotherapy, using sentinel sites’**

**S.1**

Below the reviewers can find some more details in describing the basis on how the sample size was estimated. In order to calculate sample sizes for this study, we have used in these calculations longitudinal monitoring data from the National Vertical Schistosomiasis Control Program (NVSCP) in Burkina Faso which were already collected during 2004-2006. For instance, an overall drop-out rate of 55% comparing survey 3 to survey 1 was observed in these NVSCP longitudinal monitoring data during 3 annual surveys over the course of the monitoring period and this was also incorporated in our sample size calculations. EpiSchisto was also used to get predictions within the integration era (i.e. “post-treatment environment” as there was the NVSCP before the integration) where as a starting point of the integrated programme, we used the most recent available NVSCP data at the time (from the 3^rd^ annual survey, i.e. follow-up year 2 during 2006).

The relationship between prevalence and intensity of *S. haematobium* infection was assumed to arise from a negative binomial distribution of parasites among hosts in order to estimate using maximum likelihood the inverse overdispersion (*k*) parameter required to parameterise the EpiSchisto program.

Figures 1A and 1B below illustrate these calculations, respectively, for pre-treatment baseline (2004) and 2^nd^ year post-treatment (2006) from the NVSCP longitudinal monitoring data. In addition, these figures show that k values change over time with the intervention-a finding we took into account in the sample size calculations.

***Figure S.1***

***A***

***B***

Points represent observed data of 763 Burkinabé children while the green line represents the fitted prevalence according to a negative binomial model with the overdispersion *k* parameter as a linear function of the arithmetic mean infection intensity (*m*) with expression *k*(*m*)= *k*_0_+*k*_1_*m*. Parameters for baseline were *k*_0_ = 0.050 and *k*_1_ = 0.001 and for the follow-up year 2 were *k*_0_ =0.017, and *k*_1_ = 0.0006. The figures above indicate that *k* values change over time with the intervention. This was taken into consideration in the calculations of the sample sizes.

**S.2**

Below the reviewers can find two tables that contain Pearson correlations. The first table (i.e. Table S.1) contains correlations between the average values of weather and environmental variables as derived from the 9 weather stations.

The second table (i.e. Table S.2) contains Pearson correlations between the school-level prevalences of single and dual infections (i.e. n=21) with the interpolated values of the weather and environmental variables through inverse distance weighting (i.e. assigned values to unknown points of weather and environmental variables for schools from the known average values as derived from the weather stations).

**Table S.1**

|  | **Mean altitude** | **Mean precipitation** | **Mean max temp** | **Mean min temp** | **Mean av temp** | **Mean air pressure** |
| --- | --- | --- | --- | --- | --- | --- |
| **Mean altitude** | 1 |  |  |  |  |  |
| **Mean precipitation** | 0.410  p=0.273 | 1 |  |  |  |  |
| **Mean max temp** | **-0.711**  **p=0.032** | **-0.825**  **p=0.006** | 1 |  |  |  |
| **Mean min temp** | -0.435  p=0.241 | **-0.677**  **p=0.045** | 0.650  p=0.058 | 1 |  |  |
| **Mean av temp** | -0.359  p=0.342 | **-0.885**  **p=0.002** | **0.800**  **p=0.010** | **0.875**  **p=0.002** | 1 |  |
| **Mean air pressure** | -0.639  p=0.064 | -0.284  p=0.459 | 0.623  p=0.073 | -0.047  p=0.905 | 0.096  p=0.806 | 1 |

*Statistically significant at the 0.05 significance level

**Table S.2**

|  |  |  |  |
| --- | --- | --- | --- |
|  | ***S. haematobium* prevalence◙** | **Active trachoma prevalence◘** | **Prevalence of co-infections with *S. haematobium* and active trachoma□** |
| **Air pressure◊** | 0.413  p=0.063 | **-0.762**  **p<0.001** | 0.331  p=0.143 |
| **Av temperature◊** | **0.543**  **p=0.011** | -0.399  p=0.073 | 0.342  p=0.129 |
| **Max temperature◊** | **0.688**  **p<0.001** | **-0.693**  **p<0.001** | 0.424  p=0.055 |
| **Min temperature◊** | **0.440**  **p=0.046** | -0.384  p=0.086 | 0.274  p=0.230 |
| **Precipitation◊** | **-0.631**  **p=0.002** | **0.470**  **p=0.031** | **-0.436**  **p=0.048** |
| **Altitude◊** | **-0.595**  **p=0.005** | **0.771**  **p<0.001** | -0.411  p=0.064 |

◊ Interpolated values of environmental variables.

**◙** *S. haematobium* prevalence at the school level (single infections with *S. haematobium* and co-infections with active trachoma are included in these calculations).

**◘** Active trachoma prevalence at the school level (single infections with active trachoma and co-infections with *S. haematobium* are included in these calculations).

**□** Only co-infections with *S. haematobium* and active trachoma at the school level are included in these calculations.

**S.3**

Scatter plots are displayed below to show values for the interpolated values of the environmental variables and the school-level prevalences of single and dual infections. Initially six scatter plots are displayed for the *S. haematobium* prevalences at the school level and the interpolated values of the environmental variables; then another six scatter plots are displayed between the active trachoma prevalences at the school level and the interpolated values of the environmental variables. Finally six scatter plots are displayed for the prevalences of co-infections with *S. haematobium* and active trachoma at the school level and the interpolated values of the environmental variables.

Points at all the six scatter plots above show *S. haematobium* prevalence at the school level (single infections with *S. haematobium* and co-infections with active trachoma are included in these calculations) versus the interpolated values of the environmental variables.

Points at all the six scatter plots above show active trachoma prevalence at the school level (single infections with active trachoma and co-infections with *S. haematobium* are included in these calculations) versus the interpolated values of the environmental variables.

Points at all the six scatter plots above show prevalences of co-infections at the school level (single infections are not included in these calculations) versus the interpolated values of the environmental variables.
